# A Literature Review on the Impact of the Gut Microbiome on Cancer Treatment Efficacy, Disease Evolution and Toxicity: The Implications for Hematological Malignancies

**DOI:** 10.3390/jcm14092982

**Published:** 2025-04-25

**Authors:** Ioana Gabriela Dumitru, Samuel Bogdan Todor, Cristian Ichim, Claudiu Helgiu, Alina Helgiu

**Affiliations:** Faculty of Medicine, “Lucian Blaga” University of Sibiu, 550024 Sibiu, Romania; ioanagabriela.dumitru@ulbsibiu.ro (I.G.D.); claudiuhelgiu@yahoo.com (C.H.); alinahelgiu@yahoo.fr (A.H.)

**Keywords:** gut microbiome, cancer therapy, dysbiosis, hematological malignancies, immunotherapy, probiotics

## Abstract

The gut microbiome plays a crucial role in modulating the efficacy and toxicity of cancer therapies, particularly in hematological malignancies. This review examines the dynamic interplay between gut microbiota and cancer treatments, such as chemotherapy, immunotherapy, and hematopoietic stem cell transplantation (HSCT). Disruptions in the gut microbiome, known as dysbiosis, are associated with adverse effects like gastrointestinal toxicity, neutropenia and cardiotoxicity during chemotherapy. Conversely, the supplementation of probiotics has shown potential in mitigating these side effects by enhancing gut barrier function and regulating immune responses. In HSCT, a higher diversity of gut microbiota is linked to better patient outcomes, including reduced graft-versus-host disease (GVHD) and improved survival rates. The microbiome also influences the efficacy of immunotherapies, such as immune checkpoint inhibitors and CAR-T cell therapy, by modulating immune pathways. Research suggests that certain bacteria, including *Bifidobacterium* and *Akkermansia muciniphila*, enhance therapeutic responses by promoting immune activation. Given these findings, modulating the gut microbiome could represent a novel strategy for improving cancer treatment outcomes. The growing understanding of the microbiome’s impact on cancer therapy underscores its potential as a target for personalized medicine and offers new opportunities to optimize treatment efficacy while minimizing toxic side effects.

## 1. Introduction

The human microbiome, especially the gut microbiome, significantly influences the body’s response to various treatments, including those for hematological malignancies. The human gut microbiota (GM) consists of a community of microorganisms residing within the intestinal tract, such as bacteria, archaea, fungi, protozoa and viruses. The main bacterial groups in the microbiome consist of Gram-positive Firmicutes and Gram-negative Bacteroidetes [1,2]. Moreover, these microorganisms generate metabolites, such as short-chain fatty acids (SCFAs), including acetate, propionate and butyrate, which may possess anti-carcinogenic properties by being involved in modulating histone deacetylases [3,4,5].

Human microbiota imbalance, known as dysbiosis, has been associated with various pathological conditions. In hematology, dysbiosis may contribute to the development and progression of both non-neoplastic disorders (such as iron deficiency anemia, thrombocytopenia, thrombocytosis and thrombotic conditions) and hematologic malignancies (including leukemias, lymphomas and multiple myeloma). Moreover, dysbiosis can influence the efficacy of treatments such as iron supplementation, chemotherapy, immunotherapy and hematopoietic stem cell transplantation, ultimately impacting patient outcomes [6]. Moreover, in geriatric individuals, a reduction in SCFAs promotes age-related inflammation in the intestines; not only are SCFAs one of the most important metabolites for cell metabolism, but they also facilitate the generation of extrathymic regulatory T (Treg) cells and cytokine production [7,8].

Recent advancements in biological techniques such as 16S eRNA sequencing have significantly enhanced our understanding about the relationship between human microbiota and hematological disorders, allowing research to identify unculturable bacteria by analyzing their genetic material [9]. It involves polymerase chain reaction (PCR) amplification of the 16S rRNA genes that are then sequenced using technologies such as Illumina sequencing, allowing the sequences to be grouped into Operational Taxonomic Units (OTUs) and compared to reference databases [10].

Moreover, the medical community is increasingly focusing on the gut microbiota and its ability to change the chemical structure of xenobiotics. The latest research has demonstrated the impact of gut microbiota on the processing of xenobiotics, which could have significant implications for the treatment of various diseases in the future, especially in the hematological field [11].

The link between microbial modulation and the emergence of hematological disorders will be explored, attempting to integrate and discuss recent research on how gut microbiota can be utilized in the study of hematological pathologies, emphasizing their potentially therapeutic benefits.

To conduct this narrative review, a comprehensive literature search was performed using three major scientific databases: PubMed, ScienceDirect, and Scopus. The search aimed to identify relevant studies exploring the relationship between the gut microbiome and cancer therapies, with a particular focus on hematological malignancies.

The following keywords and MeSH terms were used in various combinations:

“gut microbiome”, “microbiota”, “cancer therapy”, “chemotherapy”, “immunotherapy”, “hematopoietic stem cell transplantation”, “HSCT”, “CAR-T cells”, “immune checkpoint inhibitors”, “probiotics”, “dysbiosis”, “gastrointestinal toxicity”, “neutropenia”, “cardiotoxicity”, “graft-versus-host disease”, and “hematological malignancies”. Clinical and preclinical studies, randomized controlled trials, cohort studies, systematic reviews, and relevant narrative reviews were considered eligible for inclusion.

## 2. Mechanisms of Microbiome Influence on Treatment Response

### 2.1. Drug Metabolism

It is important to recognize that the gut microbiota can influence xenobiotic metabolism and drug responses both directly and indirectly, impacting drug efficacy and toxicity. Moreover, the gut microbiota plays a crucial role in reductive metabolism, facilitating various biochemical reactions such as acetylation, deacylation, decarboxylation, dehydroxylation, demethylation and dehalogenation. Notably, in certain cases of drug-related toxicity, it also promotes conjugate hydrolysis reactions. Beyond its direct effects, the gut microbiota exerts indirect influences on drug metabolism by modulating host metabolic pathways and competing with bacterial-derived metabolites for xenobiotic processing [12].

**Figure 1:**
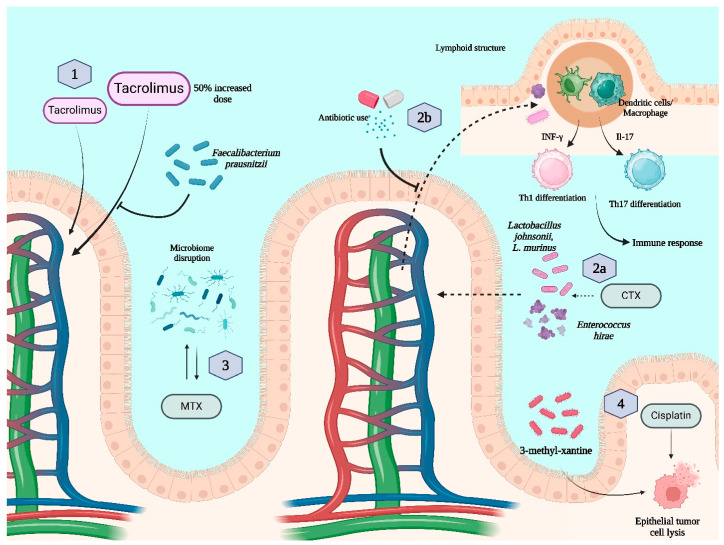
Microbiota interactions with pharmacokinetics and pharmacodynamics. 1—Faecalibacterium prausbitzii increases the necessary dose of Tacrolimus due to inactivation; 2a—cyclophosphamide (CTX) induces Enterococcus hirae and Lactobacillus species translocation to lymphoid structures and enhances immune response; 2b—antibiotics block bacterial translocation, leading to a reduced response to CTX; 3—methotrexate (MTX) and disrupted microbiome flora have a reciprocal influence; 4—3-methyl-xantine-producing flora leads to enhanced cisplatin toxicity through dopamine receptor D1 (observed only in epithelial tumors, not in lymphoma).

For example, Tacrolimus, commonly used for graft-versus-host disease (GVHD) prophylaxis in allogeneic stem cell transplantation (allo-HSCT), has a narrow therapeutic index, making it essential to understand the factors influencing its concentration for both efficacy and safety. Research has suggested that gut microbiota play a significant role in determining the required dosing of Tacrolimus. This hypothesis emerged from observations that post-transplant complications, such as diarrhea, enterocolitis and antibiotic use, are linked to fluctuations in Tacrolimus blood levels. A small study involving 19 transplant recipients found that patients with higher levels of a specific gut bacterium in their stool (*Faecalibacterium prausnitzii*) during the first post-transplant week required a 50% higher Tacrolimus dose within the first month of treatment [13] (Figure 1).

Furthermore, research has shown that cyclophosphamide, an inactive prodrug with alkylating and immunosuppressive properties, not only promotes TH1 and TH17 cellular responses and the production of IFNγ and IL-17 but also alters gut microbiota composition in mice [14,15,16]. Specifically, it facilitates the translocation of certain Gram-positive bacteria, such as *Lactobacillus johnsonii*, *Lactobacillus murinus* and *Enterococcus hirae*, to peripheral lymphoid organs in over 40% of cases. This migration triggers the production of pathogenic T helper 17 (pTH17) cells and memory TH1 cells. Interestingly, mice treated with antibiotics exhibited resistance to cyclophosphamide, likely due to a diminished pTH17 response [17,18]. Additionally, further studies have demonstrated that certain Gram-positive bacteria, including *Enterococcus hirae* and *Barnesiella intestinohominis*, enhance the antitumor effects of cyclophosphamide, highlighting the crucial role of gut microbiota in modulating its therapeutic efficacy [19] (Figure 1).

Methotrexate (MTX) is the first-line treatment for rheumatoid arthritis (RA) and widely used in non-Hodgkin lymphomas. Emerging evidence suggests that variations in the gut microbiome influence MTX bioavailability and treatment response. Studies have identified specific microbial metagenomic differences in RA patients that may predict MTX efficacy, leading to the development of machine learning models for response prediction. Additionally, MTX itself modifies the gut microbiome in a dose-dependent manner, emphasizing the bidirectional interaction known as pharmacomicrobiology [20] (Figure 1).

Metabolomic analysis revealed increased levels of the gut-derived metabolite 3-methylxanthine in antibiotic-treated mice, which was identified as a key enhancer of cisplatin-induced apoptosis through a dopamine receptor D1-dependent pathway. However, these findings were observed in epithelial tumors, not in lymphomas [21].

### 2.2. Inflammation and Immune Modulation

The gut microbiome plays a crucial role in shaping systemic immune responses, influencing treatments like immunotherapy and chemotherapy through direct and indirect interactions with tumor cells, immune modulation and metabolic inflammation. Recent research highlights its impact on cancer progression by acting as an immune modulator [22].

The role of microbiota in tumor development has been extensively studied in gastrointestinal solid tumors, but its impact on hematological malignancies remains underexplored. Recent research has investigated gut microbiota composition in primary gastrointestinal lymphomas, analyzing stool samples from patients with primary gastric lymphoma (PGL) and primary intestinal lymphoma (PIL). Notably, patients with PIL exhibited significant differences in gut microbiota diversity, both in terms of species richness (alpha diversity) and community composition (beta diversity). Through linear discriminant analysis effect size (LefSe), researchers identified 43 microbial species with altered abundance, of which 33 were decreased in PIL cases [23]. Among these, five belonged to the *Eubacterium genus*, with *Eubacterium rectale* emerging as the most relevant due to its significant decline in abundance, suggesting a possible role in disease progression [24] (Table 1).

Further experimental studies in murine models have provided additional insights into microbiota-mediated immune regulation. One study demonstrated that *Lactobacillus johnsonii* can modulate systemic inflammation and genotoxicity by selectively reducing hepatic natural killer T (NKT) cells and pro-inflammatory cytokines such as IL-1β and IFN-γ in the liver, spleen and bloodstream. Simultaneously, the study observed an increase in anti-inflammatory cytokines, including TGF-β and IL-10, highlighting a potential protective role of certain microbial species in maintaining immune homeostasis [35].

Another report identified potential causal links between genetic predispositions in the gut microbiome and lymphoma development, highlighting specific bacterial genera with protective effects against different lymphoma subtypes. Notably, *Coprobacter*, *Alistipes*, *Ruminococcaceae* and *LachnospiraceaeUCG001* were associated with reduced risks of Hodgkin’s lymphoma, diffuse large B-cell lymphoma, follicular lymphoma and T/NK cell lymphoma, respectively, with statistical analyses confirming result reliability [25]. An explanation could be that *Coprobacter*, a key member of the phylum *Trichoderma* and a major butyrate producer, supports gut health by providing energy, maintaining anaerobic conditions, preserving barrier integrity, and inhibiting inflammation and oncogenic signaling pathways such as TGF-β and Akt/ERK [26] (Table 1).

Multiple myeloma (MM) patients exhibit an increased abundance of *Proteobacteria* and a reduced presence of *Actinobacteria*, with specific bacterial taxa such as *Streptococcus*, *Klebsiella*, *Clostridium leptum* and *Pseudomonas aeruginosa* being enriched. A selective rise in nitrogen-recycling microbiota may enhance glutamine synthesis, potentially driving myeloma progression. Additionally, *Prevotella heparinolytica* has been implicated in promoting intestinal colonization and bone marrow migration of Th17 cells, leading to IL-17 production, eosinophil activation and IL-6 release, which may contribute to inflammation-driven tumor progression [27,28]. Chronic steroid exposure altered gut microbiota composition in mice, increasing *Bifidobacterium* and *Lactobacillus* while depleting *Mucispirillum*; this shift, along with reduced IL-17 production in DXM-treated mice, suggests a microbiota-mediated role in the therapeutic effects of DXM in myeloma [36] (Table 2).

A study analyzing fecal samples from 35 patients with diffuse large B-cell lymphoma (DLBCL) revealed a lower abundance of Bacteroidetes in the DLBCL group compared to healthy controls [29]. Bacteroides play a crucial role in maintaining gut health by protecting against pathogenic microbes and reducing inflammation through the production of short-chain fatty acids (SCFAs), such as butyrate, which helps regulate intestinal inflammation [30]. Additionally, research suggests that dysbiosis in cisplatin-treated patients may lead to bacterial translocation and supplementation with Ruminococcus gnavus could help mitigate systemic inflammation by lowering serum IL-6 levels [37] (Table 2).

*Veillonella*, anaerobic Gram-negative cocci, are opportunistic pathogens whose overabundance has been linked to intestinal inflammatory diseases. Research indicates that these bacteria have immunomodulatory effects by inducing IL-6 production and activating macrophages through the LPS-TLR4 pathway [38]. Increased levels of *Veillonella* have been observed in patients with diffuse large B-cell lymphoma (DLBCL) who do not achieve a complete response, suggesting a potential role in disease progression [31].

Studies have identified a link between chronic lymphocytic leukemia (CLL) and an increased Bacteroides/Firmicutes ratio, along with reduced bacterial diversity in the gut microbiota. CLL patients also exhibit higher levels of Proteobacteria, a group of bacteria commonly associated with obesity, insulin resistance and inflammatory conditions such as ulcerative colitis and Crohn’s disease [32]. Furthermore, research has shown that lower gut microbiome diversity in CLL patients correlates with disease development. Experimental models have reinforced this association, demonstrating that mice raised in highly hygienic conditions, which limit microbiome diversity, experience faster disease progression compared to those housed under less sterile conditions [33]. Another study found that CLL patients had altered microbiomes, with reduced *Bacteroidetes* and increased *Firmicutes*, particularly in those with unmutated IGHV and high CD38 expression. The *Firmicutes/Bacteroidota* ratio was proposed as a novel microbiome signature, associated with a shorter time to first treatment, suggesting microbiota’s role in disease progression [34] (Table 1).

### 2.3. Toxicity and Side Effects

Several studies have shown that the role of gut microbiota in influencing cancer prognosis, treatment efficacy, and post-treatment results highlights a dynamic interplay between environmental determinants and host genetics. While chemotherapy remains one of the primary treatments for cancer, its severe side effects continue to significantly affect patients’ quality of life [39,40].

**Figure 2:**
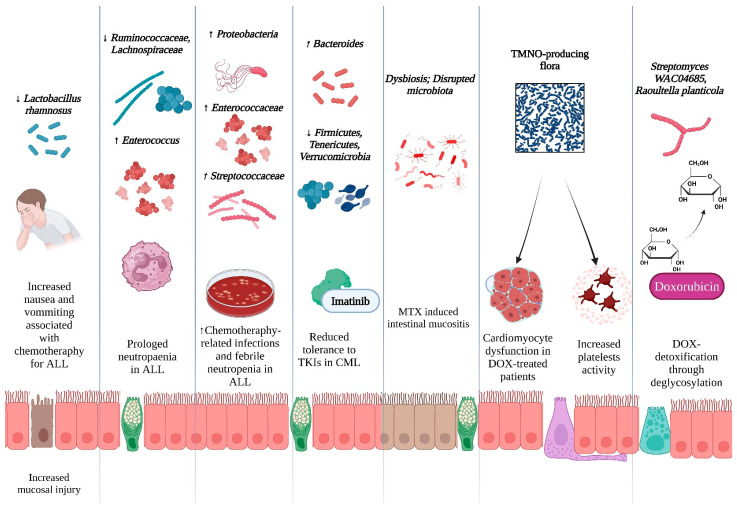
Microbiome shifts and treatment-related toxicity in hematological malignancies. Blue indicates a favorable effect, while red indicates an unfavorable effect. Upward arrows (↑) represent an increased abundance of the bacterial genus, whereas downward arrows (↓) indicate a reduced abundance.

A study explored the connection between probiotic supplementation in children with ALL receiving chemotherapy. After daily administration of *Lactobacillus rhamnosus*, a probiotic that belongs to the normal microflora, common gastrointestinal side effects such as nausea, vomiting and abdominal distension were significantly reduced. The decrease in gastrointestinal side effects could be attributed to its role in producing a biofilm that protects the gastrointestinal mucosa, as well as inhibiting Salmonella species due to lectin-like protein 1 and 2 [41,42,43] (Figure 2).

In children with newly diagnosed acute lymphoblastic leukemia (ALL), slower neutrophil recovery was linked to a decrease in beneficial gut microbiota genera, such as *Ruminococcaceae* and *Lachnospiraceae*, and an increase in *Enterococcus* overgrowth. This dysbiosis was associated with heightened chemokine signaling, indicating that an imbalance in gut bacteria may play a role in prolonged neutropenia, underscoring the importance of investigating gut-preserving approaches to support immune recovery [44] (Figure 2).

Nonetheless, a study on children with ALL found that a higher abundance of Proteobacteria in gut microbiota was associated with an increased risk of developing febrile neutropenia, whereas *Enterococcaceae* and *Streptococcaceae* predicted a greater risk of diarrhea in these patients [45]. Furthermore, a small study of 46 patients with chronic myeloid leukemia (CML) treated with tyrosine kinase inhibitors (TKIs) used 16S rRNA sequencing to analyze gut microbiota composition. It was concluded that patients with a higher abundance of *Firmicutes*, *Tenericutes* and *Verrucomicrobia* experienced fewer side effects compared to those with a greater abundance of *Bacteroides* during therapy [46] (Figure 2).

Chemotherapy drugs, such as methotrexate (MTX), can lead to intestinal mucositis, which causes a range of complications like inflammation, pain and diarrhea. Probiotics, synbiotics and related agents have shown potential in modulating intestinal microbiota to alleviate these effects. These agents regulate dysbiosis, improve epithelial barrier integrity, reduce oxidative stress and modulate the host immune response [47].

Doxorubicin (DOX) is an effective anticancer drug, but its use is limited by cardiotoxicity, which is increasingly linked to alterations in gut microbiota composition and function, known as dysbiosis, through the gut–microbiota–heart (GMH) axis. These alterations lead to an overproduction of TMAO (trimethylamine N-oxide) [48]. Additionally, it has been shown that TMAO can affect contractile function and intracellular calcium processing in cardiomyocytes, likely due to reduced energy production caused by TMAO-induced mitochondrial dysfunction [49]. Furthermore, studies have indicated that the gut microbiota can influence platelet hyperresponsiveness and blood clot formation through TMAO production, linking microbial metabolites to both cardiac and vascular health [50] (Figure 2).

Proteasome inhibitors (PIs), such as bortezomib and carfilzomib, are commonly used to treat multiple myeloma (MM) but are associated with gastrointestinal (GI) toxicity, including diarrhea. While there is limited evidence directly linking PIs to changes in gut microbiota composition, it is suggested that inhibition of the NF-κB pathway may play a role in exacerbating GI toxicity. Additionally, an imbalance in gut microbiota, such as a reduction in beneficial bacteria or an overgrowth of pathogenic bacteria, may contribute to the severity of these adverse reactions [51].

Another report found that *Streptomyces WAC04685* inactivates the anticancer drug doxorubicin through a deglycosylation mechanism involving NADH dehydrogenase, offering potential for novel drug detoxification applications [52]. Moreover, *Raoultella planticola* is a potent inactivator of doxorubicin under anaerobic conditions, demonstrating that its deglycosylation of the drug reduces toxicity and could inform clinical dosing to optimize therapeutic outcomes while minimizing side effects [53] (Figure 2).

## 3. Specific Impact on Hematological Treatments

### 3.1. Allogeneic Hematopoietic Stem Cell Transplantation (HSCT)

Hematopoietic stem cell transplantation (HSCT) following intensive marrow-ablative chemoradiotherapy has become a critical therapeutic option for various hematological, neoplastic and congenital diseases [54]. However, complications arising from the procedure contribute significantly to morbidity and mortality, accounting for 45% of deaths unrelated to relapse. Major causes of mortality after HSCT include infections, relapse and graft-versus-host disease (GVHD) [55]. Studies have shown that high levels of the *Enterococcus* genus are associated with higher rates of GVHD and mortality, while individuals with a lactose malabsorption genotype are at an increased risk for GVHD, likely due to lactose serving as a substrate for *Enterococcus* growth [56]. Additionally, increased abundance of commensal bacteria from the Blautia genus in the intestinal microbiota is associated with reduced GVHD-related mortality and improved overall survival following allogeneic blood/marrow transplantation, with factors such as antibiotic treatment and prolonged total parenteral nutrition linked to a loss of *Blautia* [57].

In a murine study, it was found that mice with lower levels of GPR109A, a G-protein coupled T-cell receptor that binds to short-chain fatty acids (SCFAs) to maintain homeostasis, had a reduced risk of developing graft-versus-host disease [58]. At high concentrations (>1 mM), butyrate induces T cell death independently of GPR109A of T cells and other immune cells. Depending on its concentration, butyrate can trigger FAS-mediated apoptosis or upregulate Th1-associated factors such as IFNγ and T-bet, independent of G protein receptors [59]. In the context of GVHD, butyrate acts as a survival factor for enterocytes and protects nonhematopoietic target tissues from GVHD in a dose-dependent manner through GPR43 [60,61].

The relationship between microbiota diversity and mortality in patients undergoing HSCT has been examined, with 16S rRNA gene sequencing of stool samples from 1362 patients. The findings showed that patients with higher microbiota diversity had a lower risk of death, with 104 deaths occurring among 354 patients in the higher-diversity group compared to 136 deaths among 350 patients in the lower-diversity group. Further analysis revealed that exposure to antibiotics such as piperacillin–tazobactam and meropenem was associated with decreased diversity during HSCT [62]. This reduction in diversity may be due to the impact of these antibiotics, which lower the abundance of bacterial groups like *Bifidobacteriales* and *Clostridiales*, leading to a decrease in butyrate production [63,64].

Research has highlighted the emerging role of multidrug-resistant (MDR) beneficial bacteria, such as *Bacteroides fragilis*, *Ruminococcus gnavus* and *Turicibacter*, in maintaining gut microbiome diversity and enhancing immune surveillance, which may help mitigate the risk of graft-versus-host disease (GvHD) in pediatric acute lymphoblastic leukemia (ALL) patients undergoing hematopoietic stem cell transplantation (HSCT). On the other hand, strains of *Enterococcus faecium* carrying multiple mutations have been linked to decreased microbial diversity and an increased risk of GvHD [65]. Further studies have suggested that the donor microbiome also plays a significant role in the development of acute GvHD, with higher bacterial diversity in the donor being associated with a lower risk of acute gastrointestinal GvHD in transplant recipients. Additionally, recipients with lower fecal bacterial diversity have been shown to have higher mortality rates, underscoring the importance of the donor’s microbiota composition in influencing HSCT outcomes [66].

### 3.2. Chemotherapy

Chemotherapy is a commonly used treatment for cancer, and over the past two decades, there have been significant improvements in the outcomes of cancer patients, particularly those with hematological malignancies. This progress is largely attributed to more aggressive chemotherapy and radiotherapy, often combined with bone marrow or stem cell rescue, as well as advancements in supportive care [67]. While the interactions between cancer and the microbiome are still not fully understood, growing attention is being paid to the potential therapeutic role of the cancer microbiome in the context of precision cancer care [68].

A report examining chemotherapy-induced dysbiosis involved patients with non-Hodgkin’s lymphoma who underwent the same myeloablative conditioning treatment without additional concurrent treatments, such as antibiotics. Two fecal samples were collected from each participant: one before chemotherapy and another seven days after chemotherapy, just prior to hematopoietic stem cell transplantation (HSCT). The results showed marked differences in the bacterial communities between pre- and post-chemotherapy samples. Alpha diversity was notably reduced in samples taken after chemotherapy and the abundance of certain beneficial bacteria, such as *Ruminococcus*, *Oscillospira*, *Clostridium* and *Bifidobacterium*, significantly decreased. In contrast, there were increases in *Citrobacter*, *Klebsiella*, *Enterococcus*, *Megasphaera* and *Parabacteroides* in post-chemotherapy samples compared to those collected before chemotherapy [69] (Table 3).

Acute leukemia patients undergoing intensive chemotherapy develop severe gut dysbiosis, with repeat therapy (re-induction or salvage) further exacerbating microbial imbalance, leading to significant *Enterococcus* expansion. This increased ecosystem instability during repeat therapy may impair colonization resistance, making patients more vulnerable to microbial overgrowth, highlighting the need for microbiota restoration therapies before or after chemotherapy cycles [70]. Furthermore, a report found that oral lactoferrin supplementation during induction chemotherapy in pediatric patients with hematologic malignancies safely promoted gut microbiome homeostasis, maintained diversity and prevented the overgrowth of harmful bacteria like *Enterococcus*, suggesting its potential as an adjunct to current therapeutic strategies [71] (Table 3).

A study identified key bacterial species involved in the immunomodulatory effects of cyclophosphamide (CTX), revealing that the Gram-negative bacterium *Barnesiella intestinihominis* plays a crucial role in anticancer immunity within the colon. Notably, the antitumor effects of CTX were enhanced by the oral administration of *Enterococcus hirae*, underscoring the significance of restoring a diverse microbiota, particularly with *Enterococcus* and *Barnesiella* species, to improve the therapeutic response to alkylating agents [19].

### 3.3. Immunotherapy

The development of immune checkpoint inhibitors (ICIs) marks a significant breakthrough in cancer treatment [80]. Unlike cytotoxic chemotherapy and small molecule inhibitors, which directly target cancer cells, ICIs boost the anti-tumor immune response by disrupting co-inhibitory signaling in T cells [81]. Research on gut microbiota has revealed that microbial components interact with immune cells through Toll-Like Receptors (TLRs), potentially playing a crucial role in enhancing antitumor immunity. Certain bacterial types have been found in patients undergoing treatment with different checkpoint inhibitors, indicating a potential shared microbial factor [82] (Table 3).

Research has emphasized the significance of the PD-1/PD-L1 pathway in cancer treatment, particularly in a murine model of acute myeloid leukemia. The interaction between PD-L1, a protein present on tumor cells, and PD-1 receptors on activated T cells results in the downregulation of T cell activity, with higher PD-L1 levels being associated with worse outcomes [83]. Additionally, the oral administration of *Bifidobacterium Bifidum* has demonstrated therapeutic effects in patients undergoing immune checkpoint inhibitor (ICI) therapy. This bacterium aids in the activation and maturation of dendritic cells and promotes the production of IFN-γ [84]. Furthermore, *Bifidobacterium Bifidum* shows potential in autoimmune disease treatment by modulating regulatory CD4+ T cells (Tregs), which play a vital role in immune regulation [85] (Table 3).

Research on lymphoma patients undergoing immune checkpoint inhibitor (ICI) therapy revealed a higher representation of *Actinobacteria* compared to healthy individuals, alongside a reduced abundance of genera such as *Bifidobacterium*, *Parabacteroides*, *Odoribacter*, *Blautia*, and *Clostridium* and a slightly increased presence of *Streptococcus*. The study also showed that patients with a positive response to treatment, either complete or partial, had a greater abundance of certain bacteria, particularly from the *Lachnospiraceae* family. In contrast, patients whose disease remained stable or progressed exhibited higher levels of Proteobacteria and Enterobacteriaceae, with a trend towards increased *Lactobacillaceae*, especially *Lactobacillus* [72]. The findings suggest that dysbiosis contributes significantly to the development and progression of B-cell lymphoma. Notably, *Lachnospiraceae* bacteria produce short-chain fatty acids (SCFAs), such as butyrate, which may enhance anti-tumor activity, while *Lactobacillus* has been recently associated with increased chemotherapy-related side effects [73] (Table 3).

An interesting study by Routy and colleagues found a significant association between the oral supplementation of *Akkermansia muciniphila* and improved efficacy of immune checkpoint inhibitors (ICIs) in drug-resistant tumor models, with the effect mediated through an interleukin-12-dependent pathway [74] (Table 3).

### 3.4. CAR-T Cell Therapy

Chimeric Antigen Receptor (CAR)-T cell therapy has revolutionized cancer treatment, delivering highly effective and durable clinical outcomes. This innovative approach involves engineering immune effector cells (IECs), like T cells and natural killer cells, to express chimeric antigen receptors (CARs), offering a new therapeutic avenue for patients with hematological malignancies. Recent research has demonstrated that CAR-T cell therapy results in remarkable remission rates for patients with relapsed hematological cancers, including acute lymphoblastic leukemia, non-Hodgkin lymphoma, chronic lymphocytic leukemia and multiple myeloma [86].

In the past decade, the role of the microbiota in CAR-T cell therapy response and toxicity has gained increasing attention. A study conducted by Smith et al. explored the impact of the gut microbiome on both the efficacy and safety of CAR-T cell therapy. Through 16S rRNA sequencing, they found that specific species of *Clostridia* were associated with achieving a complete response by day 100 following CAR-T treatment [75]. Additionally, a study explored the relationship between gut microbiota and patients with multiple myeloma (MM) undergoing CAR-T cell therapy. Dysbiosis in some MM patients may be linked to renal insufficiency, a common symptom of MM [76] (Table 3).

One study showed a significant decrease in bacterial diversity after CAR-T therapy, with *Firmicutes* becoming more abundant and *Bacteroidetes* decreasing. Moreover, there was an increase in *Enterococcus*, *Lactobacillus and Actinomyces*, while *Bifidobacterium* and *Lachnospira* levels decreased. Importantly, the study showed that lower levels of *Bifidobacterium* were correlated with more severe cytokine release syndrome (CRS), a common toxic effect of CAR-T therapy affecting approximately 80% of patients [77,78] (Table 3).

The relationship between the gut microbiome and the efficacy of CAR-T cell therapy in patients with B-cell lymphoma was explored in one study. It was found that patients who received high-risk antibiotics, such as meropenem, cefepime, ceftazidime, and piperacillin–tazobactam, during therapy exhibited higher levels of *Prevotella*, *Veillonella* and *Enterococcus* species, which were associated with poorer treatment responses. In contrast, patients who did not receive these high-risk antibiotics had higher abundances of beneficial bacteria, including *Roseburia*, *Bifidobacterium*, *Lactobacillus* and *Eubacterium species* [79] (Table 3).

## 4. Superimposed Effects of Antibiotic Therapy on Gut Microbiota in Patients with Hematological Malignancies

In addition to the effects of chemotherapy and hematological malignancies on the gut microbiota, the use of antibiotics in treating patients with hematological malignancies also plays a crucial role in shaping the microbiome. Antibiotic treatments, commonly administered to prevent or treat infections in these patients, can induce shifts in microbial diversity and composition, potentially influencing treatment outcomes and patient health.

Although neither third-generation cephalosporins (3GC) nor piperacillin/tazobactam (TZP) significantly impacted alpha diversity, both antibiotics induced distinct shifts in beta diversity, particularly in the abundance of *Enterobacteriaceae* and *Enterococcaceae* in endotracheal and perineal microbiota, suggesting that short-term antibiotic exposure can alter the microbial composition without immediately affecting overall diversity [87] (Table 4).

Doxycycline was associated with a significant short-term decrease in the alpha diversity of Bifidobacterium, while clarithromycin led to a reduction in both the numbers and diversity of Enterobacteria and anaerobic bacteria such as *Bifidobacterium* sp. and *Lactobacillus* sp. for up to 5 weeks. On the other hand, phenoxymethylpenicillin, nitrofurantoin, and amoxicillin had minimal impact on alpha diversity. As for beta diversity, antibiotics induced significant shifts in the microbial composition between individuals, with some studies suggesting that these changes could last from 2 to 6 months [88] (Table 4).

Tigecycline significantly reduces alpha diversity, with marked decreases in *Bacteroidetes* and increases in *Proteobacteria*, and this disruption persisted even five weeks after treatment cessation. It also altered beta diversity, shifting the microbial composition in a way that increased susceptibility to *Clostridioides difficile* infection despite the drug’s intrinsic activity against the pathogen [89] (Table 4).

In a cohort of 60 patients with hematological malignancies, exposure to broad-spectrum beta-lactams (BSBLs) significantly reduced alpha diversity, while levofloxacin exposure was associated with increased alpha diversity and a lower risk of dominance by non-Bacteroidetes genera [90]. Moreover, combinatorial exposure to colistin and amoxicillin significantly disrupted the alpha and beta diversity of the human intestinal microbiota, with only partial recovery observed after fecal microbiota transplantation (FMT) [91].

Single-dose administration of carbapenems (meropenem, imipenem, and ertapenem) significantly disrupted alpha diversity, as evidenced by changes in Shannon index values correlated with prolonged CRKP colonization and bacterial shedding. All three carbapenems also induced distinct shifts in beta diversity, altering the overall composition of gut microbiota and reducing colonization resistance, with ertapenem showing a comparatively milder impact [92] (Table 4).

## 5. Conclusions

The gut microbiome plays a pivotal and multifaceted role in shaping the outcomes of cancer treatments, particularly in hematological malignancies. It influences not only the effectiveness of therapies such as chemotherapy, immunotherapy and CAR-T cell therapy but also the toxicity and side effects associated with these treatments. The intricate interplay between the microbiome and cancer therapies underscores the potential for microbiome modulation as a therapeutic strategy to optimize patient outcomes.

Furthermore, interventions aimed at preserving or restoring a healthy gut microbiome, such as probiotic or antibiotic stewardship, have shown promise in mitigating side effects like gastrointestinal distress, neutropenia and infections. However, the complexity of the microbiome’s role in cancer treatment highlights the need for personalized approaches to leverage this knowledge, balancing the modulation of gut bacteria to optimize therapeutic responses while minimizing adverse effects.

## Figures and Tables

**Table 1:** Microbiome impact on hematological malignancies’ disease evolution.

Hematological Malignancy	Microbiota Alteration	Implications	Reference
Primary Intestinal Lymphoma	↓ Gut microbiota diversity, ↓ *Eubacterium rectale*	Reduced microbial diversity may contribute to disease progression	[23,24]
Gastrointestinal Lymphomas	Gut microbiota composition shifts	Altered microbiota diversity	[23]
Lymphoma	Protective effect: ↑ *Coprobacter*, *Alistipes*, *Ruminococcaceae*, *Lachnospiraceae*	Reduced risk of lymphoma subtypes through anti-inflammatory pathways	[25,26]
Hodgkin Lymphoma	↑ *Coprobacter* (protective effect)	*Coprobacter* maintain gut health and inhibit inflammatory pathways	[25]
Multiple Myeloma	↑ *Proteobacteria* (e.g., *Klebsiella*, *Pseudomonas aeruginosa*), *↓ Actinobacteria*	MM progression through affecting glutamine metabolism and inflammation	[27,28]
Diffuse Large B-Cell Lymphoma	*↓ Bacteroidetes*, *↑ Veillonella*	Reduced SCFA production linked to inflammation. *Veillonella* abundance may impact response to treatment	[29,30,31]
Chronic Lymphocytic Leukemia	↑ *Bacteroides/Firmicutes ratio*, *↑ Proteobacteria*	Dysbiosis correlates with disease progression and inflammatory conditions	[32,33]
CLL and Microbiome Signature	↑ *Firmicutes/Bacteroidota* ratio in high-risk patients	Proposed as a novel biomarker for disease progression and treatment initiation	[34]

**Table 2:** Microbiome changes and treatment response in hematological malignancies.

Hematological Treatment	Microbiota Alteration	Implications	Reference
Cyclophosphamide Treatment	↑ *Lactobacillus johnsonii*, *L. murinus*, *Enterococcus hirae* translocation	Enhances TH1 and TH17 responses, improving antitumor immunity	[35]
Dexamethasone Therapy in MM	↑ *Bifidobacterium*, *Lactobacillus*, *↓ Mucispirillum*	Modulating DXM’s effects on IL-17 production and immune response	[36]
Cisplatin-Induced Dysbiosis	↑ *Ruminococcus gnavus* supplementation lowers IL-6	Mitigate inflammation and bacterial translocation in treated patients	[37]

**Table 3:** Specific impact of microbiome shifts in hematological malignancy treatments.

Microbiota Alteration	Hematological Treatment	Impact	Reference
Increased *Enterococcus*	Allogeneic HSCT	Associated with higher GVHD and mortality	[56]
Higher *Blautia* abundance	Allogeneic HSCT	Reduced GVHD-related mortality, improved survival	[57]
Reduced microbiota diversity	Allogeneic HSCT	Higher mortality risk, worsened outcomes	[62]
Increased *Enterococcus* expansion	Chemotherapy	Gut dysbiosis, worsened ecosystem stability, increased microbial overgrowth risk	[70]
Decreased beneficial bacteria (*Ruminococcus*, *Oscillospira*, *Clostridium*, *Bifidobacterium*)	Chemotherapy	Reduced gut diversity, impaired microbiome stability	[69]
Increased *Citrobacter*, *Klebsiella*, *Enterococcus*, *Megasphaera*, *Parabacteroides*	Chemotherapy	Post-chemotherapy dysbiosis, higher infection risk	[69]
Lactoferrin supplementation	Chemotherapy	Prevents Enterococcus overgrowth, maintains gut microbiome homeostasis	[71]
Increased *Barnesiella intestinihominis*	Chemotherapy (CTX)	Enhances anticancer immunity, improves therapy response	[19]
Increased *Enterococcus hirae*	Chemotherapy (CTX)	Boosts immunomodulatory effects of therapy	[19]
Increased *Actinobacteria*	Immunotherapy (ICIs)	Observed in lymphoma patients, potential link to therapy response	[72]
Higher *Lachnospiraceae*	Immunotherapy (ICIs)	Enhanced anti-tumor activity, better response	[73]
Increased *Lactobacillus*	Immunotherapy (ICIs)	Linked to higher chemotherapy-related side effects	[73]
*Akkermansia muciniphila* supplementation	Immunotherapy (ICIs)	Improved response to immune checkpoint inhibitors (ICIs) via IL-12-dependent pathway	[74]
Increased *Clostridia* species	CAR-T Cell Therapy	Associated with better treatment response	[75]
Dysbiosis in multiple myeloma patients	CAR-T Cell Therapy	Linked to renal insufficiency, worsened therapy response	[76]
Reduced bacterial diversity post-CAR-T	CAR-T Cell Therapy	Higher Firmicutes, lower Bacteroidetes, increased Enterococcus and Lactobacillus	[77]
Lower *Bifidobacterium*	CAR-T Cell Therapy	Correlated with severe cytokine release syndrome (CRS)	[78]
Increased *Prevotella*, *Veillonella*, *Enterococcus* (with high-risk antibiotics)	CAR-T Cell Therapy	Poorer treatment response	[79]
Higher *Roseburia*, *Bifidobacterium*, *Lactobacillus* (without high-risk antibiotics)	CAR-T Cell Therapy	Better treatment response	[79]

**Table 4:** Impact of commonly used antibiotics on alpha and beta diversity in infectious complications of hematological malignancies.

Antibiotic	Alpha Diversity	Beta Diversity	Reference
3GC (Third-Generation Cephalosporins)/TZP (Piperacillin/Tazobactam)	No significant change	Significant compositional shifts, ↑ Enterobacteriaceae and Enterococcaceae	[87]
Doxycycline	↓ Bifidobacterium diversity (short-term)	Not specified	[88]
Clarithromycin	↓ Diversity and numbers of Enterobacteria, *Bifidobacterium* sp., *Lactobacillus* sp.	Changes could persist up to 5 weeks	[88]
Tigecycline	↓ Overall diversity, ↓ Bacteroidetes, ↑ Proteobacteria	Persistent shifts, ↑ CDI susceptibility	[89]
BSBL (Broad-Spectrum Beta-Lactams)	↓ Shannon index	Not specified	[90]
Levofloxacin	↑ Alpha diversity	↓ Dominance of non-Bacteroidetes genera	[90]
Colistin + Amoxicillin	↓ Diversity, incomplete recovery after FMT	Significant shifts, partially restored with FMT	[91]
Meropenem/Imipenem/Ertapenem	↓ Alpha diversity (Shannon index correlated with prolonged CRKP colonization)	Distinct compositional changes; Ertapenem had milder microbiome disruption	[92]

## Data Availability

The original contributions presented in this study are included in the article. Further inquiries can be directed to the corresponding author.
